# Isolation of intragenic suppressor of *tsp-15*-splicing mutant in *Caenorhabditis elegans*

**DOI:** 10.17912/micropub.biology.000126

**Published:** 2019-07-23

**Authors:** Hiroki Moribe, Eisuke Mekada

**Affiliations:** 1 Department of Biology, Kurume University School of Medicine, Kurume, Fukuoka, Japan; 2 Department of Cell Biology, Research Institute for Microbial Diseases, Osaka University, Suita, Osaka, Japan

**Figure 1:**
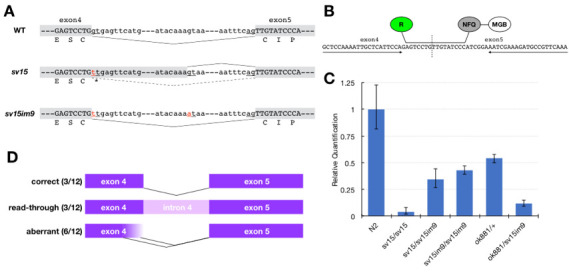
(A) Representation of the position of the *sv15* and *im9* mutations in *tsp-15*, and the effects of the mutations on the splicing of intron 4. The *sv15* mutation alters the splice donor site, and a cryptic splice donor in the middle of intron 4 is often used (Moribe *et al.* 2004)*.* The intragenic *im9* mutation abolishes the cryptic donor site, resulting in reuse of the original donor site and partial recovery of the wild-type transcript. (B) Design of qRT-PCR analysis using TaqMan probe for detecting correctly spliced *tsp-15* transcript. The positions of primers and TaqMan MGB probe are indicated. R, reporter; NFQ, nonfluorescent quencher; MGB, minor groove binder. (C) Relative expression levels of correct *tsp-15* transcripts. Data are mean RQ value and error bars indicate RQmin/RQmax (confidence set at 95%). *ok881* is a null allele of *tsp-15* having a deletion in the *tsp-15* coding region (Moribe *et al.* 2012). (D) Representation of splice pattern of *tsp-15* cDNA from *sv15im9*. Among 12 *tsp-15* cDNA subclones, we found 3 correctly spliced products, 3 containing read-through of intron 4, and 6 aberrant transcripts using -GT- in exon 4 as a cryptic splice donor.

## Description

TSP-15 is one of the 21 tetraspanins in *Caenorhabditis elegans* and is essential for exoskeletal (cuticle) development. A reduction in the function of TSP-15 results in a dumpy (Dpy) and/or blistered (Bli) phenotype (Moribe *et al.* 2004), and TSP-15 null mutants exhibit embryonic lethality (Moribe *et al.* 2012). *sv15* is a hypomorphic allele of *tsp-15*, featuring a splicing error mutation in *tsp-15*’s fourth intron. The mutation results in the immediate production of a stop codon (Fig. 1A), and the resulting truncated form of TSP-15 protein has no function. *sv15* mutants are viable despite severe cuticle deficiencies since some correctly spliced transcripts are produced (Moribe *et al.* 2004).

To further understand the functions of TSP-15 *in vivo*, we screened genetic suppressor mutants of *tsp-15(sv15)*. *tsp-15(sv15)* worms were mutagenized with 50 mM ethyl methanesulfonate (EMS) for 4 h and F_2_ progeny were screened for wild-type appearance. Nine suppressor alleles (*im1* to *im9*) were isolated from a nonclonal screen from 15,000 haploid genomes. One dominant and eight recessive suppressor alleles were included among them. The alleles were mapped by single-nucleotide polymorphism (SNP) mapping using Hawaiian variant CB4856 and characterized by DNA sequence analysis. Mutations were confirmed by complementation test, rescue by DNA transformation, and RNAi analysis. We identified one dominant allele as described here and four of eight recessive alleles, which will be described elsewhere.

The dominant allele *im9* was revealed to feature an intragenic mutation in intron 4 of *tsp-15* (Fig. 1A). *sv15/sv15im9* showed the wild-type appearance with a very mild Dpy phenotype, and the appearance of *sv15im9/sv15im9* homozygotes almost matched of the wild-type. *im9* mutation abolished a cryptic splice donor site, which is used in *sv15*, suggesting that the original splice site was possibly reused in *sv15im9* mutants. To test this hypothesis, we performed quantitative RT-PCR (qRT-PCR). To detect the correct *tsp-15* transcript, we designed a TaqMan MGB probe specific for the border between exons 4 and 5 (Fig. 1B). The *tsp-15* primer set and TaqMan probe were designed by Primer Express software (Applied Biosystems). Total RNA and first-strand cDNA were prepared as described previously (Moribe *et al.* 2004). TaqMan real-time PCR was performed and analyzed using the ABI 7500 system (Applied Biosystems). RNA polymerase II subunit *ama-1* was used as an internal control to normalize the *tsp-15* relative value, and its primers and probe were purchased from TaqMan Gene Expression Assays (Assay ID Ce02462726_m1). The PCR reaction was performed using TaqMan Universal PCR Master Mix (Applied Biosystems), in accordance with the manufacturer’s protocol. Because the amplification efficiencies of *tsp-15* and *ama-1* were almost equal, we calculated the relative amount of *tsp-15* by the comparative C_T_ method. The relative value was determined from the mean of duplicate experiments. The results showed that the correct transcript was produced from the *sv15im9* allele at a high level. *sv15im9* homozygotes were comparable to *tsp-15* null heterozygotes (*ok881/+*), which showed the wild-type appearance, in terms of the expression level of the correct transcript of *tsp-15* (Fig. 1C). This suggested that the *tsp-15* expression level in *sv15im9* was sufficient to restore exoskeletal development. In addition, we also sequenced *tsp-15* cDNA subclones isolated from *sv15im9* by RT-PCR, and confirmed the presence of correctly spliced transcripts (Fig. 1D).

The *C. elegans* splice site consensus sequences match those of vertebrates; *C. elegans* introns also obey the GU-AG rule (Blumenthal and Steward 1997). The splicing that occurred in *sv15im9* mutants did not conform to this rule, but several instances in which the splice site did not always follow the rule have already been reported. Intron recognition involves the A+U richness of introns, which is probably significant for recognition of 5′ splice sites by U1 snRNP (Blumenthal and Steward 1997).

## Reagents

**Strains:**

OB83 *tsp-15(sv15/sv15im9)*

OB119 *tsp-15(sv15im9/sv15im9)*

OB176 *tsp-15(ok881/sv15im9)*
